# Health E Englewood Health and Wellness Program: A Social Determinants of Health Intervention in Englewood, New Jersey

**DOI:** 10.7759/cureus.39646

**Published:** 2023-05-29

**Authors:** Sharmela Brijmohan, Veronica R Jacome, Mekesha Samuel, Cindy Varona, Jennifer Yanowitz, Dipal Patel, Natasha Rastogi

**Affiliations:** 1 Internal Medicine, Englewood Health and Medical Center, Englewood, USA; 2 Internal Medicine, Englewood Hospital, Englewood, USA; 3 Public Health, Englewood Hospital, Englewood, USA; 4 Internal Medicine, Engelwood Hospital, Englewood, USA; 5 Internal Medicine, Englewood Hospital and Medical Center, Englewood, USA

**Keywords:** virtual workshop, health wellness, covid 19, zoom classes, social determinant of health

## Abstract

Background: The Health E Englewood Health and Wellness Program is a social determinant of health (SDoH) intervention developed to address social factors affecting the health of the North Hudson Community Action Corporation (NHCAC) patients’, a Federally Qualified Health Center located in Englewood, New Jersey. The main aim of this integrated wellness approach was to educate and motivate participants from the local community by strengthening the development of healthy lifestyles and providing the necessary tools for positive behavior change.

Methods: Health E Englewood was a four consecutive week workshop series focused on three areas of health: physical, emotional, and nutritional wellness. The program targeted Spanish-speaking patients from NHCAC and was offered virtually via Zoom in Spanish.

Results: The Health E Englewood program was launched in October 2021 with 40 active participants. About 63% of participants attended at least three of the four workshop sessions, with at least 60% of participants reporting improved lifestyle changes after the program. Additional follow-up data collected six months later also indicated evidence of the program's long-term benefits.

Discussion: Social factors are the primary drivers of health outcomes. While many determinant interventions have failed to show long-lasting benefits, studying these interventions and their impact is crucial as it avoids “re-creating the wheel” inside health care and increasing costs.

## Introduction

The World Health Organization (WHO) defines social determinants of health as “the conditions in which people are born, grow, work, live, and age, and the wider set of forces and systems shaping the conditions of daily life. These forces and systems include economic policies and systems, development agendas, social norms, social policies, and political systems” [1]. The Health E Englewood Health and Wellness Program is a social determinant of health (SDoH) intervention developed to address social factors affecting the health of patients of the North Hudson Community Action Corporation (NHCAC), a Federally Qualified Health Center located in Englewood, New Jersey. Our clinic serves a largely underserved and uninsured community with high rates of chronic comorbid health conditions. Successfully managing these health conditions in a vulnerable population can be challenging for doctors due to complex factors in the physical and social environments that affect their patients’ health.

In the United States (US), efforts to improve health have traditionally focused on improving the healthcare system. However, studies have shown that health outcomes are largely determined by a range of factors, including genetics, health behaviors, social and environmental factors, and health care [2]. In a study cited by the National Academy of Medicine, medical care contributed only 10-20% to people's health [3]. Contrary to this, social determinants of health play a significantly greater role in influencing a person's health, accounting for 80-90% of the contributing factors [3]. Based on a meta-analysis of over 50 studies, the Kaiser Family Foundation researchers concluded that social factors, including education, racial segregation, social support, and poverty, contributed to over a third of all U.S. deaths in a year [4].

There are many developed countries that spend more on social services than the United States. Despite significant spending, U.S. health outcomes are among the lowest for developed countries [5]. Moreover, the US economy loses an estimated $309 billion annually due to the direct and indirect costs of disparities [6].

The main aim of this integrated wellness approach was to educate and motivate participants from the local community by strengthening the development of healthy lifestyles and providing the necessary tools for positive behavior change. Our hope is that addressing more upstream social determinants will improve health outcomes, reduce inequities, and lower costs.

## Materials and methods

**Figure 1:**
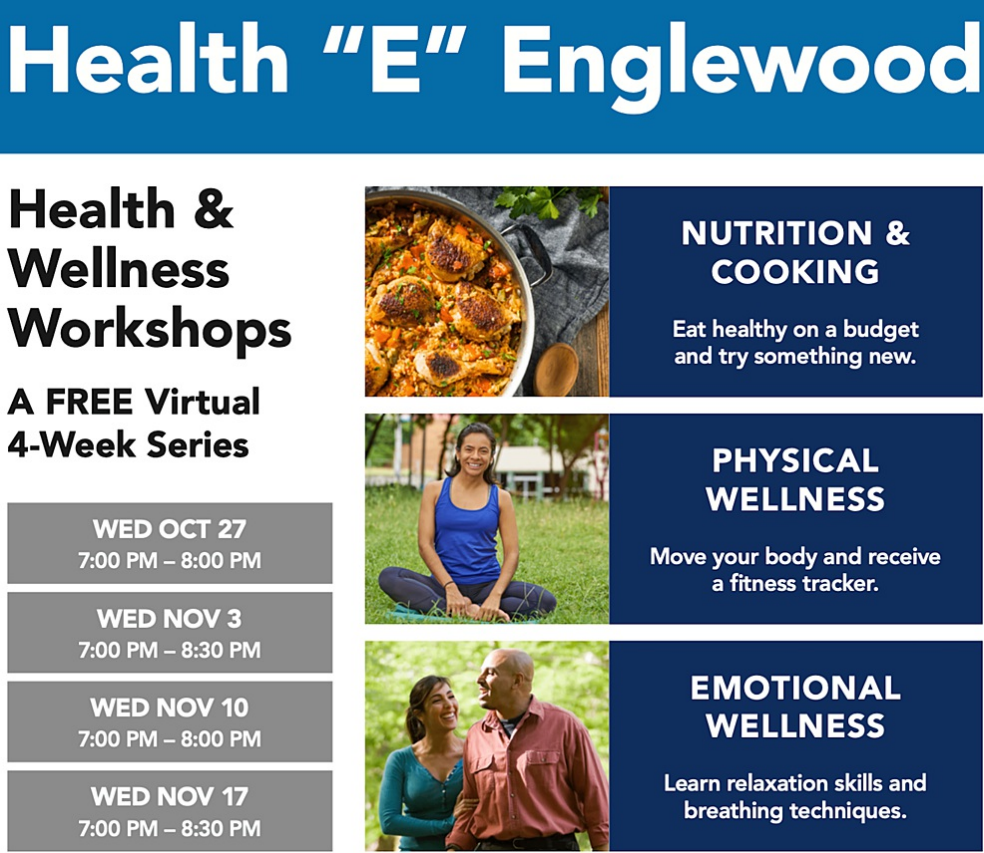
Outline of the health and wellness workshop series.

Health E Englewood was a four consecutive weeks workshop series focused on three areas of health: physical, emotional, and nutritional wellness. Each of the four sessions was built upon skills to promote healthy living, including tips to get active, practical nutrition guidance, stress management skills, and budget-friendly healthy cooking tips (Figure 1, Table 1).

**Table 1:** Descriptions of each workshop series.

Sessions	Activity
1. Physical fitness	Get fit while you sit: learn techniques to de-stress and stay fit through simple body movements and exercises you can do at home
2. Nutrition	Receive a fitness monitor and operating instructions. Better understand how to read a food label and learn tips for how to prepare healthy meals for a family on a budget.
3. Emotional wellness	Explore individual stress and how we deal with it. Develop a set of tools to help manage the stress in our daily lives
4. Cooking demonstration with chef and nutritionist	Family-style meal and gift card distribution. Healthy cooking with a chef
5. Survey completion	Track survey completion after the program ends
6. Follow-up survey at six months after the intervention	Phone calls to participants

The program targeted Black and Hispanic patients from NHCAC and was offered virtually via Zoom in Spanish and English. The recruitment of patients during routine clinic visits was coordinated by the population health team, medical assistants, and medical residents, who utilized a screening questionnaire for this purpose. Patients were eligible for the study if they expressed willingness to participate in weekly online workshop sessions conducted through Zoom and had access to a smartphone or computer with internet access to engage in the program. The resulting composition of the study comprised approximately 70% Hispanic individuals and 30% African Americans/Blacks.

**Figure 2:**
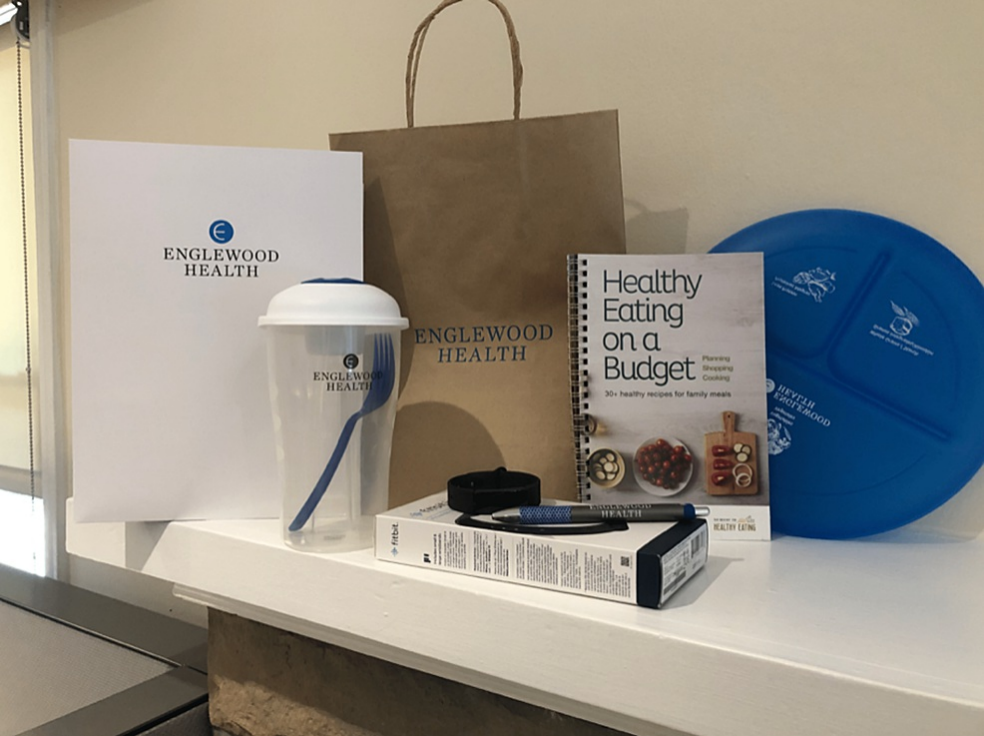
Welcome package provided to participants.

All participants received a welcome package that included Fitbit and Fitbit instructions, a portion plate, a healthy eating cookbook, a stress ball, a yoga mat, and Zoom information packets. All materials needed for the program were provided at no cost and delivered to patients’ homes (Figure 2).

Following the completion of the study, an immediate post-intervention survey was conducted to evaluate the efficacy of the interventions and gather participant feedback. Patients were then followed up after a six-month period. They were called via telephone, using a Spanish interpreter if needed. To assess the continued impact of the intervention and gather suggestions for its improvement, a similar post-intervention survey was administered to evaluate whether patients continued to benefit from the study.

## Results

**Figure 3:**
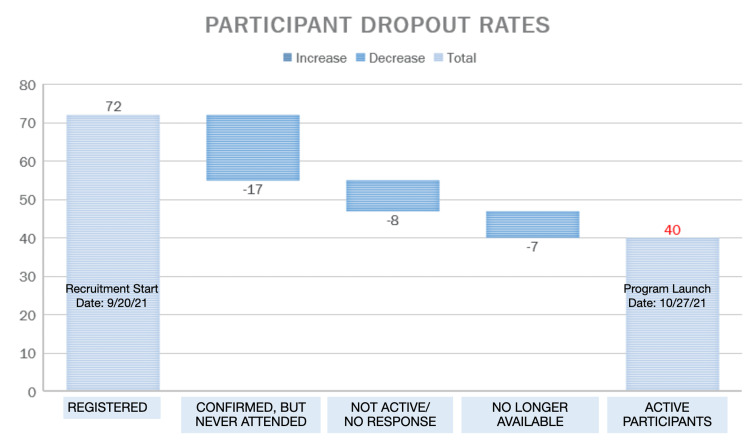
Program participants.

**Figure 4:**
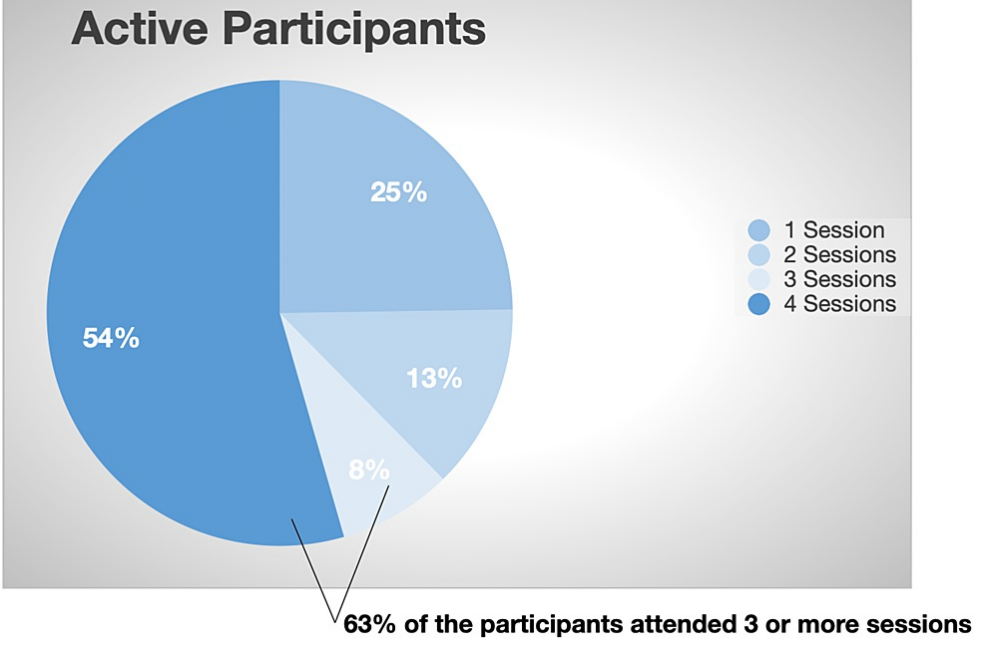
Program attendees.

The Health E Englewood program was initiated in October 2021, with a total of 72 recruited participants, out of which 40 actively participated in the program. Among the participants, 63% attended a minimum of three out of the four workshop sessions. After a period of six months, a follow-up survey was conducted, with the participation of 15 patients. See Figures 3, 4. 

**Table 2:** Shows a comparison of participants’ responses on the immediate post-intervention survey and six months after the intervention.

An immediate post-intervention survey showed the following results	On a follow-up survey six months after the program, we were able to contact 15 participants and the following was found:
About 70% of participants rated their ability to read a food label well after the program, 90% of participants rated their knowledge about cooking healthy as knowledgeable/very knowledgeable after the program, and 60% of participants rated their frequency of cooking a healthy meal for their families as very often/always after the program.	About 50% of participants still used food labels to make decisions before buying food very often/always, 70% of participants reported being able to cook healthier meals often/always, 90% of participants still used the healthy eating on a budget cookbook, 70% found it to be very helpful, 80% of participants still used the blue portion plate they were given, and 80% found it very helpful.
About 60% of participants reported feeling equipped to handle their stress after the program.	About 70% of participants continued to use the stress management techniques they were taught, and 90% found these techniques very helpful.
About 100% of participants used the Fitbit tracker and found them useful, 100% of participants increased their physical activity after receiving a Fitbit tracker.	About 100% of participants claimed to exercise regularly as part of self-care, but only 33% reported still using the Fitbit tracker. Only 10% believed the device increased their activity level.
About 100% of participants were likely to make healthy lifestyle changes after the program.	About 90% of participants believed that the knowledge and skills they learned during the program continue to influence their healthy lifestyle choices.

## Discussion

The Health E Englewood program targeted a vulnerable population of the NHCAC Clinic, with more than 50% of participants reporting improved lifestyle changes after the program and on follow-up six months after the intervention. Many similar programs have shown impressive benefits. For example, a long-term follow-up of patients registered in the ‘anticipatory care model’ revealed that this approach to medical care reduced the mortality rate in Glyncorrwg compared to a similar neighboring community in Europe [7]. On the contrary, the evidence is mixed, with some care models showing little to no benefits. For instance, the ‘Camden care model’ developed by the Camden Coalition of Healthcare Providers failed to achieve decreased readmission rates due to the diverse medical and social needs of the participants [8].

Another comparable investigation was undertaken by Alvear-Vega and Acuña San-Martín, delving into the impartial access to health care services through the Explicit Health Guarantees Plan (GES) of the Chilean population. Their findings indicate that the GES Plan effectively fulfills its primary objective of providing support to socially vulnerable groups with lower socioeconomic status. However, the study also revealed significant gaps in healthcare access among patients with certain medical conditions. These findings highlight the need for targeted interventions to ensure equitable access to healthcare services for all individuals, regardless of their specific medical conditions [9].

The Health E Englewood program participants were recruited by the population health team, medical assistants, and medical residents using a screening questionnaire. This intervention was not a one-size-fits-all approach but was instead pioneered to be culturally competent with personalized guidance for barriers encountered [10]. We believed we were able to retain many of our participants by providing interval reminders through phone calls, text messages, financial gift card incentives, building trust, and customizing the program to patients' needs. The intervention was also unique in that it was done virtually due to the global pandemic, a time in which there was significant disruption to our social lives, physical activity, and mental health. Hassan et al. also conducted a study utilizing web-based technology to address social determinants of health. Their findings revealed that this approach has the potential to significantly reduce the need for additional team members, thus minimizing costs, while also enabling widespread dissemination of the intervention [11].

While fine-tuning our intervention for a particular population proved beneficial in the immediate post-intervention period, additional follow-up data collected six months later also indicated evidence of the long-term benefits of the program. In the six-month follow-up survey, most participants believed that lack of time and motivation were the major obstacles to implementing what they have learned. Healthy cooking for a family was hindered by time constraints and individual meal preferences. However, many believed the portion plate was helpful as it was a tangible reminder of healthy portion size. As an unintended benefit, participants also built their own social network, in which they continued to share ideas even after the end of the program [12].

A similar program, which encompassed a therapeutic lifestyle modification (TLM) program, was implemented with a six-month follow-up period. This program focused on women residing in a community in Korea. The TLM program consisted of health monitoring, counseling, health education, exercise, and dietary interventions. The researchers discovered that the positive outcomes of the program, particularly reductions in body weight and waist circumference, were sustained even six months after the intervention. However, similar to our intervention, it is important to note that not all aspects of the program demonstrated the same level of sustainability [13]. These findings highlight the complexity of interventions targeting social determinants of health and emphasize the need for continuous evaluation and follow-up assessments to monitor the long-term effectiveness of such programs. It is crucial to identify and understand the factors that contribute to the sustainability or lack thereof, of specific health outcomes, in order to design more comprehensive and effective interventions in the future.

As part of our future interventions, we hope to focus on meal preparation and bulk cooking to accommodate busy schedules. The use of tangible reminders, such as storage containers, might be helpful. Also, we intend to return to in-person workshops as we believe some interventions, such as the Fitbit tutorial, would be better realized in person. Ideally, we would like participants to use a whole range of these techniques rather than just a few, since we believe that only then will the program have its full impact [14].

One significant limitation of this intervention was that it had to be conducted virtually due to COVID-19 restrictions. As a result, patients who did not have access to the internet or suitable devices were unable to participate. This could have potentially excluded certain individuals who may have benefited from the program, especially those from lower-income households, elderly populations, or those residing in rural or remote areas with limited access to technology.

Another limitation was the relatively high dropout rate, with 32 enrolled participants failing to attend any of the sessions. This could have been due to various reasons, such as scheduling conflicts, lack of motivation, or technical difficulties. Identifying and addressing such barriers is essential to improve the engagement and participation of individuals who may benefit from the program [15-18].

Throughout the program, we have made extensive efforts to identify and address various barriers that may impede progress or hinder the achievement of our goals, as shown in Table 3.

**Table 3:** Barriers encountered during the program.

Presented barriers	Solutions
Cost	Program offered free of charge
Language	Bilingual support, presenters
Access	Programs offered virtually, in the evenings, pre-recorded videos
Transportation	Organized deliveries of program materials
Communication	Weekly dissemination of information through email, SMS messaging, and voice calls
Comprehension	Simplified content, the live platform provided for discussion with presenters
Program materials	Provided in Spanish
Technology	Personalized assistance provided
Trust	Professional guidance provided through culturally competent care
Continued resources	Sustainable skills and tools to incorporate in lifestyles following the ending of the program

Overall, it is crucial to recognize and address the limitations of these interventions to ensure that all potential beneficiaries have equal access to the program and maximize its impact. 

## Conclusions

Social factors are the primary drivers of health outcomes. The ideal SDoH intervention is one that decreases per capita spending and has the greatest effect on total population health and well-being. Well-designed interventions tailored to those most in need can curb the overuse of our resource-constrained system. Although these techniques may be discussed with patients during routine visits with their primary care doctors, SDoH interventions play a vital role in healthcare by providing a supportive environment for patients to learn how to implement healthy lifestyle practices.
